# Respiratory MUC5B disproportion is involved in severe community-acquired pneumonia

**DOI:** 10.1186/s12890-022-01870-x

**Published:** 2022-03-15

**Authors:** Lu Fan, Yi Lu, Yan Wang, Xiaomin Zhang, Yuxuan Wu, Hao Sun, Jinsong Zhang

**Affiliations:** 1 Department of Emergency, Jiangsu Province Hospital, The First Affiliated Hospital of Nanjing Medical University, 300 Guangzhou Rd, Nanjing, 210029 People’s Republic of China; 2 Department of Emergency, Northern Jiangsu People’s Hospital Affiliated to Yangzhou University, 98 Nantong West Rd, Yangzhou, 225001 People’s Republic of China; 3 Department of Respiratory Medicine, Qixia Branch of Jiangsu Province Hospital, 28 Yaojia Rd, Nanjing, 210033 People’s Republic of China; 4 Intensive Care Unit, Nanjing Chest Hospital, 215 Guangzhou Rd, Nanjing, 210029 People’s Republic of China

**Keywords:** Community-acquired pneumonia, MUC5B, Severity, Disproportion

## Abstract

**Background:**

Mucus production is a process involved in the pathogenesis of Community-acquired pneumonia (CAP). The study is to determine Mucin 5B (MUC5B) protein concentration and its proportion in the bronchoalveolar lavage fluid (BALF) of CAP patients and evaluate its value to help assess disease severity.

**Methods:**

A total of 118 patients were enrolled in this cross-sectional study, including 45 with severe CAP (SCAP) and 73 with non-severe CAP (NSCAP). MUC5B concentration in BALF were determined by immunoblotting analysis. Total protein concentration of BALF was detected by Pierce BCA kit. Cytokines IL6, IL10, IFNγ, IL13, and IL17 in BALF were measured using commercial enzyme-linked immunosorbent assay (ELISA). Spearman’s correlation analysis was applied to evaluate the relationships between MUC5B concentration or MUC5B/total protein ratio and the CURB-65 score, as well as cytokines. Logistic regression analysis was used to identify the independent factors associated with severe CAP. Receiver operating characteristic (ROC) curve was used to evaluate the assessment value of MUC5B/total protein ratio and other indexes for CAP severity.

**Results:**

MUC5B concentration in the BALF of NSCAP group was higher than that in SCAP group [NSCAP 13.56 µg/ml (IQR 5.92–25.79) vs. SCAP 8.20 µg/ml (IQR 4.97–14.03), *p* = 0.011]. The total protein concentration in the BALF of NSCAP group was lower than that in SCAP group [NSCAP 0.38 mg/ml (IQR 0.15–1.10) vs. SCAP 0.68 mg/ml (IQR 0.46–1.69), *p* = 0.002]. The MUC5B/total protein ratio was remarkably higher in NSCAP group than that in SCAP groups [NSCAP 3.66% (IQR 1.50–5.56%) vs. SCAP 1.38% (IQR 0.73–1.76%), *p* < 0.001]. MUC5B/total protein ratio was negatively correlated with total protein concentration (r_s_ = − 0.576, *p* < 0.001), IL6 (r_s_ = − 0.312, *p* = 0.001), IL10 (r_s_ = − 0.228, *p* = 0.013), IL13 (r_s_ = − 0.183, *p* = 0.048), IL17 (r_s_ = − 0.282, *p* = 0.002) and CURB-65 score (r_s_ = − 0.239, *p* = 0.009). Logistic regression identified that MUC5B/total protein ratio, IL6 level and CURB-65 score as independent variables related to CAP severity. ROC curve demonstrated best assessment value of MUC5B/total protein ratio for SCAP (AUC 0.803, *p* < 0.001), with a sensitivity of 88.9% and a specificity of 64.4%.

**Conclusions:**

Respiratory MUC5B disproportion is related to CAP severity. MUC5B/total protein ratio may serve as an assessment marker and a potential therapeutic target for severe CAP.

## Introduction

Community-acquired pneumonia (CAP) is one of major lethal infectious disease [1] with the incidence reaches 30–50% in adults [2]. Approximately 10% patients with CAP will develop severe CAP (SCAP) and require ICU treatment in which the mortality rate ranges from 19 to 50% [3]. Common symptoms of CAP include fever, cough and increased sputum production [4]. However, severe cases are prone to develop serious complications due to complexity and heterogeneity of variant risk factors [5], such as virulence and serotypes of pathogens, the age, immune state and comorbidities of patients, as well as genetic variants [6]. Given the inadequacy of the condition assessment of SCAP, a profound understanding of SCAP pathogenesis and a sensitive molecular marker are expected to improve patients’ diagnosis and treatment.

One major feature of CAP is mucus production and mucus is mainly composed of mucins [7, 8]. Previous animal experiments have proved that airway secreted mucin MUC5B but not Mucin 5AC (MUC5AC) plays a critical role in immune defense against bacterial infections [9]. MUC5B helps form gel in the airway as a defensive barrier and regulates the rheology of airway mucus [10]. Electron microscopy has shown that multiple MUC5B filaments always appear as strands lining in the airway. It traps and sweeps away pathogens from the lung by working with other types of mucins [11]. Previous studies showed that MUC5B participates in the development of pulmonary diseases, such as pulmonary fibrosis, chronic obstructive pulmonary disease (COPD) and bronchiectasis [12–14]. So far, whether MUC5B expression is involved in the development of SCAP has not been studied.

In our study, we compared the MUC5B concentration and its proportion in bronchoalveolar lavage fluid (BALF) of patients with NSCAP and SCAP. We analyzed the correlation between MUC5B expression and CAP severity to determine its value as a marker to help assess disease severity.

## Material and methods

### Study design

This cross-sectional study recruited patients from three hospitals including Jiangsu Province Hospital, Nanjing Chest Hospital and Qixia Branch of Jiangsu Province Hospital. The consecutive adult CAP patients treated in the respiratory and critical care general ward or intensive care unit (ICU) from Jan 2021 to Aug 2021 were screened for participants. The study was approved by the Institutional Review Board of coordinating center Jiangsu Province Hospital (No. 2021-SR-028). Written informed consent of the bronchoalveolar lavage (BAL) procedure and BALF sample utilization was obtained from each participant or from a relative or main care.

The inclusion criteria were as follows: 1. Men and women aged over 18 years old; 2. Diagnosed of CAP according the ATS guidelines; 3. Untreated or received treatment less than 24 h before admission; 4. Patients underwent BAL as standard of diagnosis [15, 16]; 5. Signed informed consent form. The exclusion criteria were as follows: 1. Patients with active pulmonary tuberculosis; 2. Patients with chronic pulmonary disease including chronic bronchitis, bronchiectasis, asthma, lung cancer, pulmonary embolism or interstitial lung diseases; 3. Patients with malignancy or severe immunosuppression; 4. Patients with pregnancy.

According to the ATS Guidelines [17] for the management of adults with CAP: CAP was diagnosed if the patient presented with at least one newly acquired respiratory symptoms such as cough, sputum and dyspnea setting on at communities, accompanied with fever, abnormal breath sounds and crackles. Each patient underwent a computerized tomography (CT) scan to identify patchy infiltrate shadows, lobar or segmental consolidation shadows, ground glass shadows or interstitial changes.

Severe CAP (SCAP) was defined during 24 h after admission by the criteria in ATS consensus guidelines 2007 [18]. The major criteria are as follows: 1. Patients needing invasive mechanical ventilation; 2. Patients showing septic shock needing vasopressors. The minor criteria included: 1. Respiratory rate ≥ 30 breaths/min; 2. PaO2/FiO2 ratio ≤ 250; 3. Multi-lobar infiltrates; 4. Confusion/disorientation; 5. Uraemia (BUN level ≥ 20 mg/dl); 6. Leucopenia (WBC < 4,000/mm^3^); 7. Thrombocytopenia (platelet count < 100,000 /mm^3^); 8. Hypothermia (core temperature < 36 °C); 9. Hypotension requiring aggressive fluid resuscitation. CAP patients presenting at least one major criterion or at least three minor criteria were diagnosed as SCAP.

Baseline clinical parameters of patients were collected from electronic hospital records (EHR). All participants received BAL procedure during 24 h after hospitalization. Through standardized bronchoscopy and performed the protocol of 2012 ATS guideline for BAL [19], the BAL was proceeded using a 120 ml lavage of 0.9% saline [20]. The BAL fluid (BALF) was then collected through a sputum box attaching to the suction canal of the bronchoscope with qualified at least 10% returned rate [21]. The BALF sample was filtered by gauze and immediately frozen at − 80 °C for further experiment. CT scan and blood test were carried out before lavage. Blood indexes included white blood cells count (WBC), leukocyte classification, lactate dehydrogenase (LDH), C-reaction protein (CRP), erythrocyte sedimentation rate (ESR), fibrinogen (Fib), and d-dimer (D-D).

### Inflammatory markers in BALF tested by ELISA

Concentration of cytokines including interleukin-6 (IL6), interleukin-10 (IL10), interferon-γ (IFNγ), interleukin-13 (IL13), and interleukin-17 (IL17) in BALF was determined using commercial enzyme-linked immunosorbent assay (ELISA) kits specific for human following protocols (IL6, Cat. #: EHC007; IL10, Cat. #: EHC009; IFNγ, Cat. #: EHC102g; IL13, Cat. #: EHC137; IL17 Cat. #: EHC170; Neobioscience; Shenzhen; China).

### MUC5B expression measurement

The BALF was thawed and then centrifuged at 1200r for 10 min at 4 °C. MUC5B in the supernatant was measured by immunoblotting analysis using S&S MINIFOLD® I and has been described in our previous study [22]. The MUC5B standard concentration driving from human saliva ranged from 0.87 to 0.01 (µg/ml). Samples were diluted with dilution buffer (3 M urea) with a ratio of 1:50. Standards and samples were run in duplicate (100 ul per sample). The protein blots binding to PVDF membrane were incubated with MUC5B primary antibodies (Santa Cruz Bio.), then with secondary goat anti-rabbit IgG Biotin conjugate and strep-HRP (Life Technologies). The blots were visualized using an enhanced chemiluminescence system. Immunoreactive dots were quantified using ImageJ software. Total protein concentration in BALF supernatant was determined by Pierce BCA protein assay kit (Cat. #: 23227; Thermo Scientific, Rockford, USA).

### Statistical analysis

The current sample size achieved the study objectives with a desired power 0.80. Statistical analyses were performed with SPSS 17.0 (SPSS Inc., Chicago, IL). Continuous variables with normal distribution were presented as mean ± SD. Variables with abnormal distribution were presented as median (Interquartile range, IQR). Categorical variables were displayed as percentages. Student t-test was applied for comparison between normal distributed variables. Mann–Whitney U test was used to compare abnormal distributed variables. The Fisher’s exact test or chi-square test was used to compare categorical variables. Spearman’s correlation coefficients (r_s_) were measured to evaluate the relationships between MUC5B concentration or MUC5B/Total protein ratio and CURB-65 score, total protein concentration, as well as inflammatory markers in BALF. Binary logistic regression analysis was used to identify independent factors associated with severe CAP. The assessment value of MUC5B/Total protein ratio or other indexes for CAP severity was evaluated using receiver operating characteristic (ROC) curve. All statistics were two-sided, only a *p* value less than 0.05 was considered statistically significant.

## Results

### Demographic characteristics and clinical parameters of participants

**Fig. 1:**
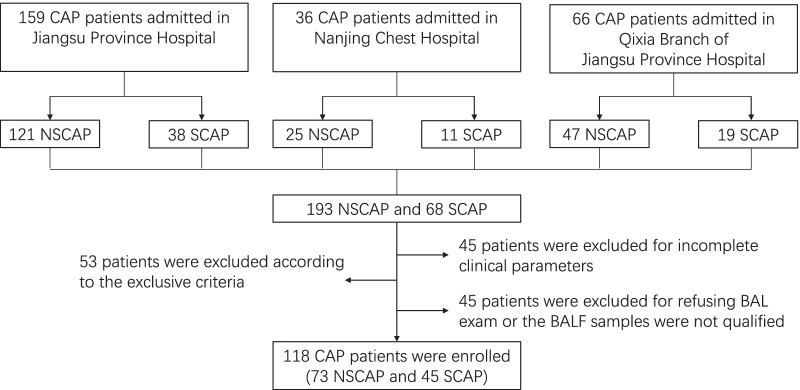
Study flowchart of patient enrollment. CAP community-acquired pneumonia, NSCAP non-severe CAP, SCAP severe CAP

A total of 118 patients confirmed with CAP were included in the research and divided into NSCAP group (n = 73) and SCAP group (n = 45) (Fig. 1). Demographic characteristics and clinical parameters are described in Table 1. The frequency of difficulty breathing was significantly higher in SCAP group than in NSCAP group (*p* = 0.001). Other symptoms including cough, sputum and fever showed no significant differences between the two groups. CURB-65 scores were significantly higher in SCAP group than that in NSCAP group (*p* < 0.001). In laboratory parameters, percentage of neutrophils (NE%), serum levels of LDH, CRP, ESR, plasma level of Fib and D-D were remarkably higher in SCAP patients than those in NSCAP patients (*p* < 0.05, respectively). Percentage of lymphocytes (LYM%) in SCAP group was lower than that in NSCAP group (*p* = 0.028).

**Table 1:** Demographic and clinical characteristics of the subjects enrolled in this study

Characteristic	NSCAP	SCAP	*p* value
*Demographics*			
N	73	45	
Age (years)	50.9 ± 14.1	56.2 ± 16.7	0.068
Gender, male (%)	42 (57.5%)	18 (40.0%)	0.064
*Symptoms and CURB-65 score*			
Cough	62 (84.9%)	40 (88.9%)	0.542
Sputum	54 (74%)	36 (80%)	0.455
Difficulty breathing	14 (19.2%)	22 (48.9%)	0.001
Fever	35 (47.9%)	24 (53.3%)	0.570
CURB-65 score			
0	57 (78.1%)	20 (44.4%)	< 0.001
1	15 (20.5%)	14 (31.1%)	
2	1 (1.4%)	8 (17.8%)	
3	0 (0%)	2 (4.4%)	
4	0 (0%)	1 (2.2%)	
5	0 (0%)	0 (0%)	
*Comorbid conditions*			
Current or ex-smoker	21 (28.8%)	6 (13.3%)	0.053
Hypertension	15 (20.5%)	15 (33.3%)	0.121
Diabetes mellitus	9 (12.3%)	6 (13.3%)	0.874
Chronic cardiac disease	1 (1.4%)	1 (2.2%)	0.728
Prior malignancy	5 (6.8%)	0 (0%)	0.073
Immune disorder	0 (0%)	1 (2.2%)	0.201
*Inflammatory biomarkers in Peripheral blood*			
WBC (10^9^/l)	6.1 (4.9–8.0)	6.5 (5.2–8.1)	0.814
NEUT (%)	64.6 (54.6–73.4)	71.0 (61.3–78.0)	0.028
LYM (%)	26.7 (18.3–35.2)	19.6 (16.4–30.0)	0.028
MO (%)	6.0 (5.0–7.3)	5.7 (4.5–7.9)	0.459
EO (%)	1.5 (0.7–2.6)	1.6 (0.7–2.4)	0.934
PLT (10^9^/l)	230 (178.5–269.5)	229 (168.5–249.5)	0.458
LDH (U/l)	108.0 (20.8–177.0)	203.0 (177.5–231.0)	< 0.001
CRP (mg/l)	3.8 (0.6–29.0)	16.0 (2.4–56.7)	0.024
ESR (mm/h)	21.0 (8.0–32.0)	56.5 (37.8–87.3)	< 0.001
Fib (g/l)	3.3 (2.4–5.1)	4.4 (3.2–5.9)	0.012
D-D (mg/l)	0.3 (0.1–0.7)	0.6 (0.4–1.2)	0.002

Data presented as number (percentage) or mean ± SD or median (interquartile range)

CAP, community-acquired pneumonia; NSCAP, non-severe CAP; SCAP, severe CAP; CURB-65, confusion, urea level, respiratory rate, blood pressure, and age > 65 years; WBC, white blood cell; NEUT%, percentage of neutrophils; LYM%, percentage of lymphocytes; MO%, percentage of monocytes; EO%, percentage of eosinophilic granulocytes; PLT, blood platelets; LDH, lactate dehydrogenase; CRP, C-reactive protein; ESR, erythrocyte sedimentation rate; Fib, fibrinogen; D-D, d-dimer

### MUC5B condition and its correlation with related indexes

MUC5B concentration in the BALF of NSCAP group was higher than that in SCAP group [NSCAP 13.56 µg/ml (IQR 5.92–25.79) vs. SCAP 8.20 µg/ml (IQR 4.97–14.03), *p* = 0.011]. The total protein concentration in the BALF of NSCAP group was lower than that in SCAP group [NSCAP 0.38 mg/ml (IQR 0.15–1.10) vs. SCAP 0.68 mg/ml (IQR 0.46–1.69), *p* = 0.002]. The MUC5B/total protein ratio was remarkably higher in NSCAP group than that in SCAP groups [NSCAP 3.66% (IQR 1.50–5.56%) vs. SCAP 1.38% (IQR 0.73–1.76%), *p* < 0.001]. For inflammatory markers in BALF, the levels of IL6, IL13 and IL17 were lower in NSCAP group than those in SCAP group (*p* < 0.05). The levels of IL10 and IFNγ showed no significant difference between the two groups (Table 2).

**Table 2:** MUC5B concentration and inflammatory biomarkers in BALF of NSCAP patients and SCAP patients

	NSCAP	SCAP	*p* value
*Muncins in BALF*			
MUC5B concentration (µg/ml)	13.56 (5.92–25.79)	8.20 (4.97–14.03)	0.011
Total protein contentration (mg/ml)	0.38 (0.15–1.10)	0.68 (0.46–1.69)	0.002
MUC5B/Total protein percent (%)	3.66 (1.50–5.56)	1.38 (0.73–1.76)	< 0.001
*Inflammatory biomarkers in BALF*			
IL6 (pg/ml)	1.81 (1.22–3.08)	5.83 (2.50–15.73)	< 0.001
IL10 (pg/ml)	0.18 (0.11–0.32)	0.15 (0.12–0.27)	0.632
IFNγ (pg/ml)	2.98 (1.90–4.04)	3.27 (2.11–4.69)	0.195
IL13 (pg/ml)	30.00 (28.78–31.66)	31.08 (30.20–33.35)	0.004
IL17 (pg/ml)	9.53 (6.52–11.23)	12.04 (9.47–17.54)	< 0.001

Data presented as median (interquartile range)

CAP, community-acquired pneumonia; NSCAP, non-severe CAP; SCAP, severe CAP; BALF, bronchoalveolar lavage fluid

The correlations among MUC5B level, total protein concentration, MUC5B/total protein ratio and the inflammatory parameters in BALF as well as CURB-65 score were investigated by Spearman’s correlation analysis (Table 3). MUC5B level had positive correlation with total protein concentration (r_s_ = 0.423, *p* < 0.001) but no correlation with inflammatory cytokines. MUC5B/total protein ratio was negatively correlated with total protein concentration (r_s_ = − 0.576, *p* < 0.001), IL6 (r_s_ = − 0.312, *p* = 0.001), IL10 (r_s_ = − 0.228, *p* = 0.013), IL13 (r_s_ = − 0.183, *p* = 0.048) and IL17 (r_s_ = − 0.282, *p* = 0.002). MUC5B level and MUC5B/total protein ratio both had negative correlations with CURB-65 score (r_s_ = − 0.218, *p* = 0.018 for MUC5B, r_s_ = − 0.239, *p* = 0.009 for MUC5B/total protein ratio, respectively).

**Table 3:** Correlation between MUC5B level, total protein concentration, MUC5B/total protein ratio and related indexes

Related indexes	MUC5B		MUC5B/total protein ratio	
	r_s_	*p* value	r_s_	*p* value
Total protein	0.423	< 0.001	− 0.576	< 0.001
IL6	− 0.021	0.820	− 0.312	0.001
IL10	− 0.095	0.307	− 0.228	0.013
IFNγ	− 0.145	0.118	− 0.044	0.636
IL13	− 0.101	0.276	− 0.183	0.048
IL17	0.085	0.360	− 0.282	0.002
CURB-65 score	− 0.218	0.018	− 0.239	0.009

r_s_, spearman rho correlation coefficients, CURB-65, confusion, urea level, respiratory rate, blood pressure, and age > 65 years

### Assessment performance of MUC5B/total protein ration for CAP severity

Though MUC5B concentration and total protein level showed significant differences between NSCAP and SCAP groups, the binary logistic regression analysis revealed that MUC5B concentration and total protein level were not independent factors associated with CAP severity. Logistic regression identified that MUC5B/total protein ratio, IL6 level and CURB-65 score remained as variables significantly related to CAP severity (Table 4).

**Table 4:** Logistic regression of variable parameters for determining the severity of CAP

Variable	OR	95% CI	*p* value
MUC5B	0.980	0.929–1.035	0.475
Total protein	0.902	0.693–1.175	0.446
MUC5B/total protein ratio	0.417	0.230–0.756	0.004
IL6	1.117	1.036–1.205	0.004
IL13	0.944	0.742–1.201	0.638
IL17	1.072	0.964–1.192	0.201
CURB-65 score	3.494	1.450–8.415	0.005
Constant	4.236		0.688

OR, Odds ratio; CI, confidence interval; CURB-65, confusion, urea level, respiratory rate, blood pressure, and age > 65 years

**Fig. 2:**
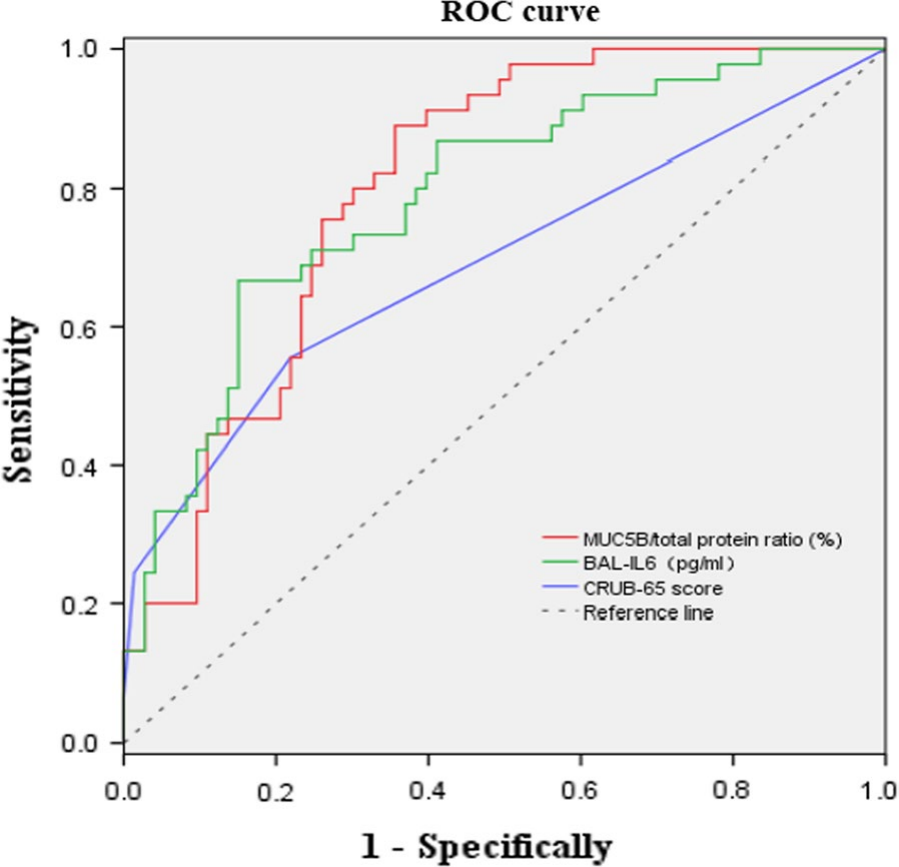
ROC curve analysis of various parameters for discriminating SCAP from NSCAP

ROC analysis was applied to evaluate whether MUC5B/total protein ratio could be used as a sensitive biomarker for CAP severity. Table 5 and Fig. 2 showed MUC5B/total protein ratio (AUC 0.803, *p* < 0.001) presented a better performance to assess the CAP severity with a sensitivity of 88.9% and a specificity of 64.4% comparing to CURB-65 score (AUC 0.692, *p* < 0.001) and the cytokine IL6 (AUC = 0.791, *p* < 0.001). The optimal cut-off point of MUC5B/total protein ratio to distinguish SCAP from NSCAP was 2.117%, with a positive predictive value of 60.6% and a negative predictive value of 90.4%.

**Table 5:** Areas under the curve of variable parameters for determining the severity of CAP

Parameter	Cut-off value	AUC	Sensitivity	Specifity	*p* value	95% CI	
						Lower limit	Higher limit
MUC5B/total protein ratio	< 2.117	0.803	0.889	0.644	< 0.001	0.726	0.880
IL6 (pg/ml)	> 4.349	0.791	0.667	0.849	< 0.001	0.708	0.874
CRUB-65 score	–	0.692	0.556	0.781	< 0.001	0.588	0.795

AUC, area under the curve; CI, confidence interval; CURB-65, confusion, urea level, respiratory rate, blood pressure, and age > 65 years

## Discussion

In the present study, we showed that the MUC5B concentration and MUC5B/total protein ratio in SCAP group were obviously lower than those in NSCAP group. The MUC5B/total protein ratio was an independent factor associated with CAP severity. We speculate that the disproportion of respiratory MUC5B plays an important role in regulating pulmonary inflammation in severe CAP.

MUC5B is the main expressed mucin in normal human and mouse airway [23, 24]. It plays a constitutive role in mucus barrier which traps and eliminates particulates and pathogens via mucociliary clearance (MCC) [25, 26]. In vivo and vitro experiments showed MUC5B expression could be induced by multiple pathogens, such as Rhinovirus (RV) [27], Mengovirus [28], Pseudomonas aeruginosa (PA) [29], Mycoplasma pneumoniae [30], A. pleuropneumoniae [31] and Pneumocystis [32]. The mechanism mainly involves STAT3-STAT6/EGFR-FOXA2 signaling [29, 30, 33]. Overexpressed MUC5B can bind to pathogens to prevent their attachment to the epithelium and initiate the clearance of airway pathogens [34, 35]. Furthermore, *Muc5b* defect increases the accumulation of pathogens in mice respiratory tract, which leads to chronic bacterial infection and hardly dissolved inflammation [9, 36]. By contrast, Muc5AC is the other secreted mucin expressed in airway, but it is not required for MCC or for diminishing infections in the airway [9]. It was concluded that MUC5B is the only secreted mucin that regulates airway homeostasis and mucosal immunity in humans [9]. In our observational study, the result showed MUC5B/total protein ratio was significantly related with CAP severity. However, whether lower concentration of MUC5B was involved in the develop of CAP severity was uncertain. We assume that MUC5B disproportion may diminish the barrier function of respiratory tract during CAP pathological process, but the hypothesis needs to be proved by further animal experiments.

Furthermore, we assessed the relationship between MUC5B and inflammatory factors. The results showed that the SCAP patients exhibited an increase in a broad scope of cytokines in BALF, especially IL6, IL13 and IL17. MUC5B/total protein ratio was negatively correlated with levels of IL6, IL10, IL13 and IL17, especially IL6 (*p* = 0.001) and IL17 (*p* = 0.002). IL6 is a pro-inflammatory cytokine involved in the pathogenesis of airway inflammatory diseases [37]. It decides CD4 + T cell fate and promotes preferential Th2 differentiation [38, 39]. In vitro study has demonstrated that IL-6 regulates Muc5b expression via the ERK signaling pathway [40]. IL-17 is also a strong pro-inflammatory factor that can induce excess inflammation through cytokine cascade [41]. It has been reported that IL-17 mediates Muc5b expression by the ERK signaling pathway and NF-kB-based transcriptional mechanism [40, 42]. MUC5B disproportion was correlated with high IL6 and IL17, indicating its contribution to lung inflammatory augmentation in severe CAP. We suggest that multiple inflammatory pathways coordinate with MUC5B disproportion regulate CAP pathogenesis.

MUC5B disproportion could also be detected in other pulmonary diseases and smoke exposure. The SPIROMICS data showed that MUC5B concentration in sputum increased with COPD severity [13]. Ever-smokers (current and former smokers) without evidence of COPD also had a higher concentration of MUC5B in sputum than no-smoke controls [43]. Our studies excluded patients with history of COPD. But the percentage of ever-smokers in NSCAP group was slightly higher than the SCAP group. It seems that smoke exposure might be partially accountable to the higher MUC5B level in NSCAP group. But when comparing the MUC5B level between ever-smokers and no-smokers, no significant difference was detected neither in the NSCAP group nor in the SCAP group. We assumed there may be limited impact of smoke exposure on the level of MUC5B. In the future we would like to conduct a randomized controlled trail to clarify this issue. MUC5B disproportion could also be found in other pulmonary diseases. In Non-CF Bronchiectasis (NCFB) study, the ratio of MUC5AC to MUC5B was about 4 times higher in NCFB patients than in healthy controls [14]. In asthma, MUC5B levels decreased or remained the same, while other types of mucin levels were increased significantly [44]. The latest reports showed abnormal MUC5B expression in COVID-19 patients [45]. Further study found MUC5B playing a protective role against COVID-19 [46]. We suggested that regulating MUC5B proportion might be a therapy target for SCAP patients in future. A recent research has proven that restoring Muc5b level can improve lung function and alleviate inflammatory responses in a rodent model [47], but more studies are needed to confirm the role of MUC5B in SCAP development.

## Limitations

First, this result was based on a small population sized cross-sectional study. The patients included in the study underwent BAL as standard of diagnosis. It may lead to a selection bias. In the further, we would like to conduct a randomized controlled trial to get results nearer to true circumstance. Second, the possible difference in MUC5B concentration between BALF and spontaneous sputum should be considered. Another issue is that we used immunoblotting to detect the concentration of MUC5B by antibody binding reaction, the accuracy of which might be affected by complex patterns of MUC5B glycosylation. These limitations will be overcome in future studies.

## Conclusions

Respiratory MUC5B disproportion is related to CAP severity. MUC5B/total protein ratio is inversely correlated with the levels of inflammatory cytokines and may serve as an assessment marker and a potential therapeutic target for severe CAP.

## Data Availability

The datasets used and analysed during the current study are available from the corresponding author on reasonable request.

## Abbreviations

CAP: Community-acquired pneumonia
SCAP: Severe CAP
NSCAP: Non-severe CAP
COPD: Chronic obstructive pulmonary disease
MUC5B: Mucin 5B
MUC5AC: Mucin 5AC
BAL: Bronchoalveolar lavage
BALF: Bronchoalveolar lavage fluid
ELISA: Enzyme-linked immunosorbent assay
ROC: Receiver operating characteristic
AUC: Area under the curve
ICU: Intensive care unit
CT: Computerized tomography
EHR: Electronic hospital records
CURB-65: Confusion, urea level, respiratory rate, blood pressure, and age > 65 years
WBC: White blood cells
NE%: Percentage of neutrophils
LYM%: Percentage of lymphocytes
MO%: Percentage of monocytes
EO%: Percentage of eosinophilic granulocytes
PLT: Blood platelets
LDH: Lactate dehydrogenase
CRP: C-reaction protein
ESR: Erythrocyte sedimentation rate
Fib: Fibrinogen
D-D: D-dimer
IL6: Interleukin-6
IL10: Interleukin-10
IFNγ: Interferon-γ
IL13: Interleukin-13
IL17: Interleukin-17
IQR: Interquartile range
CI: Confidence interval
OR: Odds ratio
r_s_: Spearman rho correlation coefficients
